# Systematic kMC Study of Doped Hole Injection Layers in Organic Electronics

**DOI:** 10.3389/fchem.2021.809415

**Published:** 2022-01-18

**Authors:** Ali Deniz Özdemir, Simon Kaiser, Tobias Neumann, Franz Symalla, Wolfgang Wenzel

**Affiliations:** ^1^ Institute of Nanotechnology, Karlsruhe Institute of Technology (KIT), Karlsruhe, Germany; ^2^ Nanomatch GmbH, Karlsruhe, Germany

**Keywords:** OLED, KMC, organic semiconductor, hole injection layer, doping

## Abstract

Organic light emitting diodes (OLED) play an important role in commercial displays and are promising candidates for energy-efficient lighting applications. Although they have been continuously developed since their discovery in 1987, some unresolved challenges remain. The performance of OLEDs is determined by a multifaceted interplay of materials and device architectures. A commonly used technique to overcome the charge injection barrier from the electrodes to the organic layers, are doped injection layers. The optimization of doped injection layers is critical for high-efficiency OLED devices, but has been driven mainly by chemical intuition and experimental experience, slowing down the progress in this field. Therefore, computer-aided methods for material and device modeling are promising tools to accelerate the device development process. In this work, we studied the effect of doped hole injection layers on the injection barrier in dependence on material and layer properties by using a parametric kinetic Monte Carlo model. We were able to quantitatively elucidate the influence of doping concentration, material properties, and layer thickness on the injection barrier and device conductivity, leading to the conclusion that our kMC model is suitable for virtual device design.

## 1 Introduction

Since their discovery in 1987 (Tang and VanSlyke, 1987) organic light emitting diodes (OLEDs) gained lots of attention in academia and industry and have been continuously developed. Modern OLEDs find application in display technology and are promising devices for efficient general lighting applications (Adachi et al., 2000; Forrest et al., 1997). Three major factors comprise the performance and usability of OLEDs: the internal quantum efficiency (IQE), the device driving voltage (Jou et al., 2015) and light outcoupling (Flämmich et al., 2011). Common strategies to improve OLED efficiency and lifetime are based on intuition and experimental trial-and-error approaches. Quantitative models of charge injection and transfer processes in OLEDs can help to systematically increase device performance and to overcome remaining issues (Lee et al., 2017; Friederich et al., 2019; Song et al., 2020). The enormous number of potential materials and device architectures turns the development of novel materials and devices into a time- and resource-intensive task. In recent years, multiscale computational methods successfully predicted charge carrier mobility in pure materials (Friederich et al., 2014; Massé et al., 2016; Kotadiya et al., 2018) and guest-host systems (Symalla et al., 2016), current voltage characteristics (Mesta et al., 2013; Kaiser et al., 2021) and photoluminescent quenching (Symalla et al., 2020b) thus gaining relevance for the organic electronics community to be used as a supporting tool in device development and optimization (Andrienko, 2018; Friederich et al., 2019). Established simulation methods to model charge transport, charge injection (extraction) in OLEDs are drift-diffusion methods (DD) (Rossi et al., 2020; Doan et al., 2019), macroscopic equivalent-circuit techniques (Nowy et al., 2010), and microscopic methods like kinetic Monte Carlo (kMC) or master equation approaches (ME) (Zojer, 2021). Despite their high computational costs, kMC based simulation methods emerge as a quantitative tool in device modeling (van der Holst et al., 2011; Symalla et al., 2018; Symalla et al., 2019). kMC simulations are capable of treating the charge hopping processes explicitly and to take into account electrostatic interactions with the surrounding charge carriers beyond a mean-field description (Casalegno et al., 2010; van der Holst et al., 2011; Casalegno et al., 2013; Liu et al., 2017). Another important advantage of kMC methods is the treatment of molecular doping where the coulomb interaction of charges located on neighboring molecular sites play a crucial role (Fediai et al., 2019; Fediai et al., 2020).

Doping of charge injection layers is an established technique to overcome obstacles like insufficient charge balance or limited charge carrier concentration in the device due to large charge injection barriers (Zhang and Blom, 2010; Chiba et al., 2017) and high driving voltages. However, understanding of these effects remains elusive. Therefore, we studied p-doping of hole injection layers (HIL) on the kMC level, focusing on: 1) the Fermi level alignment of the doped injection layer and 2) how the p-doping influences the conductivity of the device. Our results confirm that our kMC model is able to perform device and material simulations to systematically investigate the influence of doping concentration, material selection and layer thickness on Fermi level alignment and device conductivity.

## 2 Methods

We performed the simulations on systems represented by simple cubic latices (Bässler, 1993; Pasveer et al., 2005) for each organic layer with a lattice constant was *d* = 1 nm (Mesta et al., 2013). Electronic properties like the ionization potential (IP) of the host material, electron affinity (EA) of the dopants or energetic disorder were treated as parameters and could easily be replaced by data from first-principle calculations, as done in previous works (Friederich et al., 2016; Kaiser et al., 2021). Charge carrier transport, charge injection (ejection) and doping activation with the kMC package LightForge (LF) (Symalla et al., 2016). In this approach, each microscopic process inside the device is modeled as a discrete event, with a corresponding event-rate. Each lattice site *i* represents an organic molecule with predefined ionization potential $E_{i}^{\text{IP}}$ , electron affinity $E_{i}^{\text{EA}}$ and reorganization energy *λ*. The energetic disorder of the organic layer is denoted by *σ*. Charge carrier dynamics and their interactions are treated explicitly by taking into account their Coulomb interactions explicitly. The Coulomb interaction is of particular importance in modeling doping activation (Arkhipov et al., 2005).

### 2.1 Charge Injection

The injection of holes from the anode to molecular site *i* of the organic layers is considered as a discrete process modeled with the Miller-Abrahams (Miller and Abrahams, 1960) rate to account for the ability of the electrodes to dissipate continuous amounts of energy. The expression for the injection rate $\omega_{i}^{\left(\text{Inj}\right)}$ is given by:

$$\omega_{i}^{\left(\text{inj}\right)}=\frac{\pi }{2k_{\text{B}}T\hbar }|J_{i}^{\left(\text{inj}\right)}{|}^{2}\left\{\begin{array}{cc} \text{exp}\left(\frac{-\Delta E_{i}}{4k_{\text{B}}T}\right)\; & ,\;\text{for}\;\Delta E_{i}>0\\ 1\; & ,\text{ for }\Delta E_{i}\leq 0 \end{array}\right.,$$ (1)

where *k*_B_ *T* is the thermal energy, $J_{i}^{\left(\text{inj}\right)}$ is the electronic coupling of the anode with site *i*. The effective hole injection barrier is given by

$$\Delta E_{i}=E_{i}^{\text{IP}}-W-\phi_{\text{screen}}-\phi_{\text{dyn}}+eFr_{i}\;,$$ (2)

with electrode work function *W*, the electric field strength *F* and *r*_*i*_ the distance between site *i* and the electrode projected onto the field direction. To take into account the Coulomb interaction between all charge carriers in the system, we consider the dynamic Coulomb contribution to the effective injection barrier:

$$\phi_{\text{dyn}}=\frac{e^{2}}{4\pi \epsilon_{0}\epsilon_{r}}\left(\underset{j}{\sum }\frac{1}{r_{ij}}-\underset{k}{\sum }\frac{1}{r_{ik}}\right)\;,$$ (3)

where *r*_*ij*_ (*r*_*ik*_) are the distances between the site *i* and all positive (negative) charges inside the device. After each kMC-step, the dynamic Coulomb interaction is recomputed by performing an Ewald-summation (Ewald, 1921). To fulfill the boundary condition of a constant electrostatic potential on the electrode surface, the screening term $\phi_{\text{screen}}=-e^{2}{\left(16\pi \epsilon_{0}\epsilon_{r}r_{i}\right)}^{-1}$ has to be included to the injection barrier, where *r*_*i*_ is the distance of site *i* to the surface of the electrode. The remaining term in Eq. 2 is the energy due to the applied electric field *F*. The opposite process (charge carriers hop from the organic material to electrode) is modeled analogously.

### 2.2 Charge Transfer

We assume that charge carriers are localized on the individual sites and that the transport from site *i* to site *j* takes place as a hopping process with the Marcus rate (Marcus, 1956):

$$\omega_{ij}=\frac{2\pi }{\hbar }|J_{ij}{|}^{2}\frac{1}{\sqrt{4\pi \lambda k_{\text{B}}T}}exp\left(-\frac{{\left(\lambda +\Delta E_{ij}\right)}^{2}}{4\lambda k_{\text{B}}T}\right)\;,$$ (4)

where *J*_*ij*_ is the electronic coupling between the sites, *λ* the reorganization energy and Δ*E*_*ij*_ contains the difference of the site ionization potentials due to the energetic disorder, the applied electric field and the dynamic electrostatic potential like in Eq. 2. For the electronic coupling *J*_*ij*_ we use the empiric expression:

$$J_{ij}=j_{0}\exp\left(-2\frac{r_{ij}}{a_{0}}\right)\;,$$ (5)

where *j*_0_ is a constant, *r*_*ij*_ is the distance between site *i* and *j* and *a*_0_ is the coupling decay length. The values for *j*_0_ and *a*_0_ can be found in the supplementary information.

### 2.3 Doping

The doped HIL consists of host (H) and dopant (D) sites arranged in a cubic grid with the dopant sites randomly distributed in the lattice. For p-doping, the dopant extracts an electron from a host site, leading to an ionized host and negatively charged dopant molecule:

$$H+D\to H^{+}+D^{-}\;.$$ (6)

In the kMC protocol doping activation (ionization of a host/dopant pair) is treated by explicitly taking into account the Coulomb interaction *V*_C_ of the host/dopant pair and the interaction between the host/dopant pair and all other charge carriers in the device (similar to *ϕ*_screen_ discussed above). The doping activation energy reads:

$$\Delta E_{\text{ion}}=-\Delta E_{\text{off}}+V_{\text{C}}+\Delta E_{\text{ext}}\pm eFr_{\text{H}\text{D}}\;,$$ (7)

where the first term $\Delta E_{\text{off}}=E_{\text{D}}^{\text{EA}}-E_{\text{H}}^{\text{IP}}$ is the energy difference between dopant electron affinity $E_{\text{D}}^{\text{EA}}$ and host ionization potential $E_{\text{H}}^{\text{IP}}$ . The Coulomb interaction between host cation and dopant anion is given by $V_{\text{C}}=-e^{2}{\left(4\pi \epsilon_{o}\epsilon_{r}r_{\text{H}\text{D}}\right)}^{-1}$ , where *r*_HD_ is the distance between the host and dopant sites. Similar to the electrostatic interactions discussed above, the ionized host/dopant pair interacts with all charge carriers inside the devices. The contribution of the explicit Coulomb interaction is given by:

$$\Delta E_{\text{ext}}=\frac{e^{2}}{4\pi \epsilon_{0}\epsilon_{r}}\left(\underset{\alpha }{\sum }\left(\frac{1}{r_{\alpha \text{D}}}-\frac{1}{r_{\alpha \text{H}}}\right)+\underset{\beta }{\sum }\left(\frac{1}{r_{\beta \text{H}}}-\frac{1}{r_{\beta \text{D}}}\right)\right)\;,$$ (8)

where *r*_*α*(*β*)H(D)_ is the distance between the activated host (dopant) molecule and negative *α* (positive *β*) charge carriers inside the device. Since the dopants in the device are randomly distributed and charge carrier positions fluctuate strongly, Δ*E*_ext_ is a stochastic quantity and changes with each kMC step. We treat doping as charge transfer from the dopant to the host, with the doping activation rates obtained by using Equation 4 and replacing Δ*E*_*ij*_ by Δ*E*_ion_.

## 3 Results and Discussion

### 3.1 Effect of Doped Injection Layer on Voltage Drop

#### Virtual Device for Measurement of Fermi Level Alignment

**FIGURE 1:**
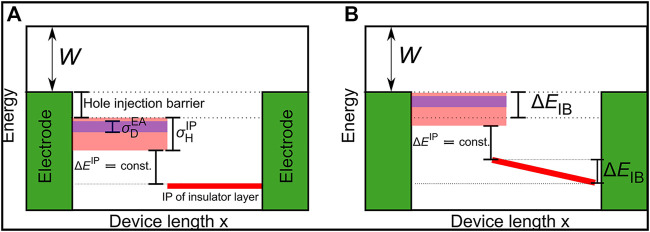
Schematic energy level diagram of the device to measure doping induced injection barrier reduction. The device consists of two identical electrodes with the workfunction *W* = 4.5 eV and two organic layers: the doped hole injection layer (HIL) and the hole blocking layer right to it. **(A)** The host ionization potential (IP) $E_{\text{H}}^{\text{IP}}$ is indicated by the light red beam with a width indicating the energetic disorder $\sigma_{\text{H}}^{\text{IP}}$ . Analogously, the dopant electron affinity (EA) with disorder $\sigma_{\text{D}}^{\text{EA}}$ is shown by the blue beam. **(B)** After activation of the host/dopant pairs free charge carriers (here holes) are ejected from the doped HIL into the anode, leaving a negative net charge in the organic layer and causing an upward shift of the energy levels, reducing the injection barrier. The energy levels are shifted until the tail states of the host IP reach the Fermi level of the electrodes.

In the first part of this study we investigated the effect of the doping concentration on the hole injection barrier between the anode and the doped HIL. The schematic structure of the simulated device is shown in Figure 1A. It consists of two organic layers where the first one is the doped HIL and the second one serves as hole blocking layer, with a layer thickness of 15 nm each. Both electrodes have the same workfunction *W* = 4.5 eV and no external voltage is applied. After activation of the host/dopant pairs free holes are ejected into the anode, leaving a negative net charge in the organic layer, causing an upwards shift of the energy levels. This process takes place until the tail states of the organic layer align with the Fermi level of the anode. Figure 1B shows the energy levels of the device after Fermi level alignment. As the energy barrier between the doped injection layer and insulating layer is constant, the Fermi level alignment causes a voltage drop in the insulating layer, which is observed in a slope of the energy levels. We compute the reduction of the injection barrier Δ*E*_IB_ as the difference in average site energies of the first and last 1 nm–slice of the insulating layer.

#### Effect of Initial Injection Barrier on Δ*E*_IB_

**FIGURE 2:**
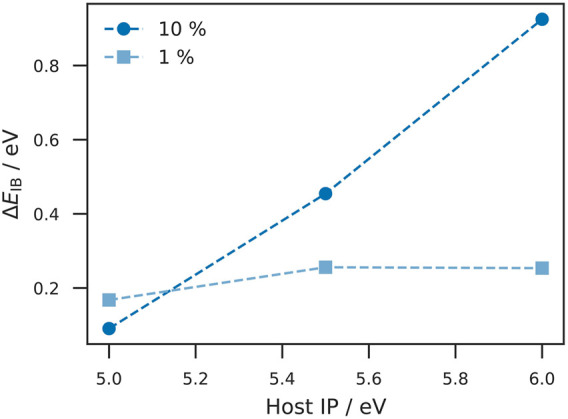
Injection barrier reduction at different host ionization potentials and doping concentrations. The reduction of the injection barrier Δ*E*_IB_ is plotted against the host IP. The squares (dots) depict a doping concentration of 1% (10%). At a host IP of 5.0  eV, the observed Δ*E*_IB_ for 10% doping concentration is smaller than for 1% which appears unexpected. However, this can be explained by considering the fact of a doping induced increase of the energetic disorder. For small initial injection barriers, the broadening of the energy levels leads to an overlap between the tail states and the electrode Fermi level, which in turn prevents further energy alignment. Nevertheless, the difference in Δ*E*_IB_ is relatively small. For larger host IPs, Δ*E*_IB_ remains almost constant at 1% doping concentration which can be explained by the fact that not enough dopants and thus free charge carriers are available to foster Fermi level alignment. At 10% doping concentration, we observe a significant increase in Δ*E*_IB_ which is explained by the presence of sufficiently enough charge carriers.

Depending on the initial injection barrier, a low doping concentration may be sufficient to reach Fermi level alignment. Therefore, we studied Δ*E*_IB_ in the insulating layer at different initial injection barriers and doping concentrations. This study was performed on the device displayed in Figure 1 at different doping concentrations and energy levels of the host and dopant sites. The difference between IP and EA was kept constant at 0.5 eV to ensure equal doping efficiencies between the simulations. The simulation was performed with *λ* = 0.2 eV for all three site types and $\sigma_{\text{H}\left(\text{D}\right)}^{\text{IP}}=\sigma_{\text{H}\left(\text{D}\right)}^{\text{EA}}=$ 0.15 eV for the host and dopant sites. The parameters for electronic couplings and the reorganization energies for charge transfer and doping activation were identical. Doping concentration has substantial impact on Δ*E*_IB_ as a function of host IP (see Figure 2). The reduction of the injecton barrier at a host IP of 5.0 eV with a doping concentration of 10% is slightly below Δ*E*_IB_ for 1% which can be explained by a doping induced broadening of the energy distribution in the doped injection layer (increase in energetic disorder) and the resulting overlap between the tail states and the Fermi level of the electrode. For larger initial injection barriers (larger host IP), higher doping concentration leads to a significant increase of Δ*E*_IB_. While at low doping concentrations there are not enough dopants and thus free charge carriers to achieve Fermi level alignment (Δ*E*_IB_ at ≈ 0.2 eV). High doping concentrations lead to a large reduction of the injection barrier and thus good Fermi level alignment even for large initial injection barriers.

#### Impact of the Device Thickness on Δ*E*_IB_

**FIGURE 3:**
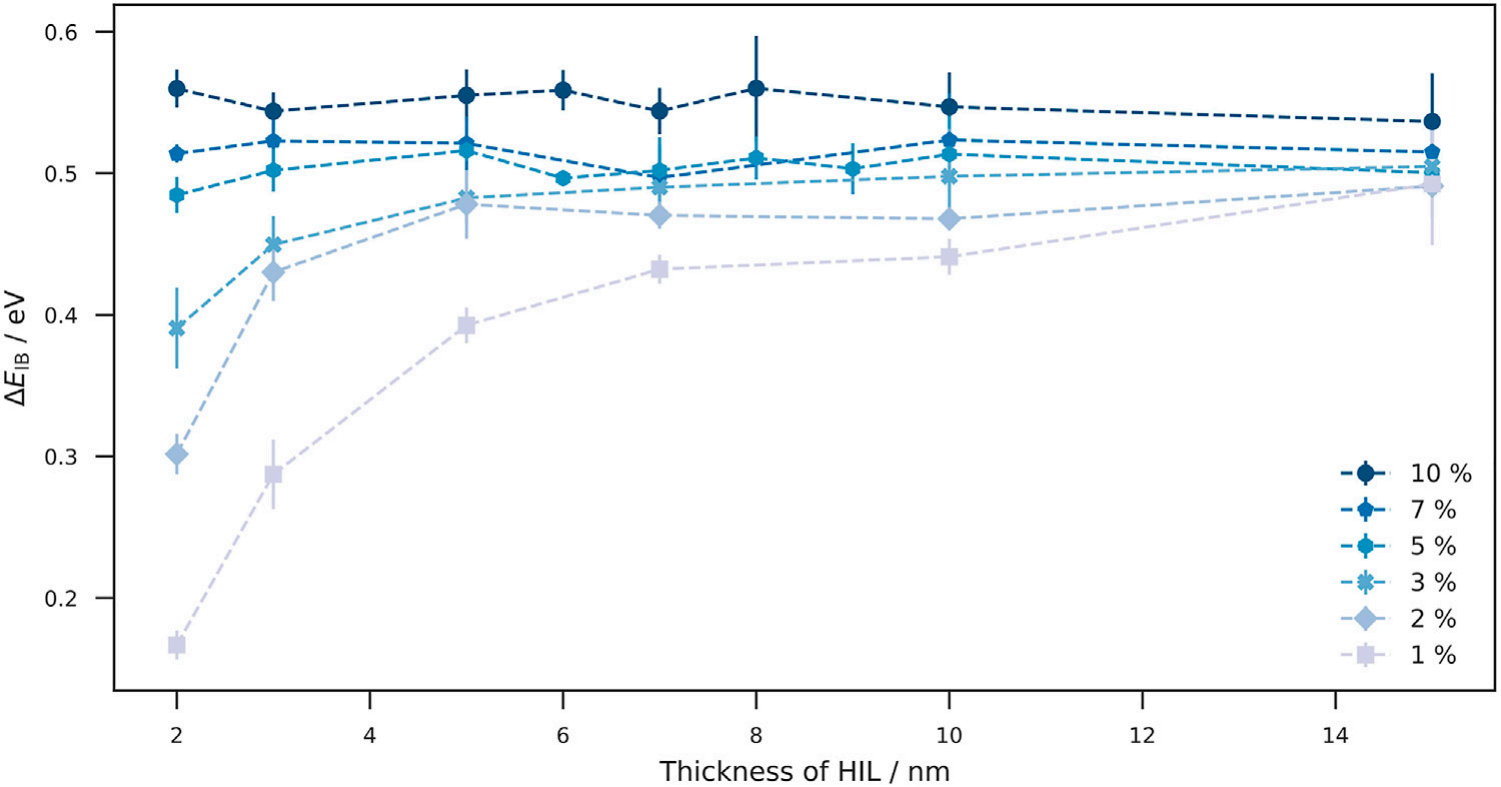
Effect of layer thickness on the **Δ*E***_**IB**_. At a given doping concentration, the thickness of the doped injection layer controls Δ*E*_IB_. While the effect of layer thickness is significant at low concentrations (1–3%), Δ*E*_IB_ at large doping concentration remains almost constant with increasing layer thickness. The weak variation of Δ*E*_IB_ at doping concentrations of 5–10% is due to the fact that sufficient dopants are already present at low layer thicknesses to provide enough charge carriers for Fermi-level alignment. A direct implication of the dependence of Δ*E*_IB_ on the total number of dopants is that, in addition to the doping concentration, the layer thickness of the doped injection layer can also be considered as a parameter for the reduction of the injection barrier. At maximum layer thickness, Δ*E*_IB_ converges to a value of about 0.5  eV, with an offset between the doping concentrations.

Besides the doping concentration, we additionally investigated the effect of the layer thickness (with constant doping concentration) on the injection barrier. Figure 3 shows Δ*E*_IB_ at different doping concentrations plotted against the layer thickness. At lower doping concentrations (1–3%), we observe how the layer thickness leads to a significant increase in Δ*E*_IB_. At a doping concentration of 1%, a high layer thickness leads to Δ*E*_IB_ from 0.1 eV to almost 0.5 eV. With increasing doping concentration, this effect becomes steadily weaker to the point where it becomes negligible: at 7 and 10% the voltage drop does not increase with the layer thickness. At a layer thickness of 15 nm, we observe a voltage drop of about 0.5 eV for all doping concentrations, with a clearly visible offset especially between the 10% curve and the others. An explanation for the offset could be the finite size effect: the site energy levels are Gaussian distributed so that the probability for host sites with tail-state energies is lower for high doping concentrations. Thus, if the tail-states are truncated, a higher energy shift is required to achieve Fermi level alignment which results in larger voltage drops in the order of the energetic disorder. These results imply, that the tendency of the doped injection layer to align with Fermi level depends on the number of intrinsically free charge carriers and thus on the number of dopants present. To increase the number of free charge carriers, there are two possibilities: increasing 1) doping concentration or 2) the layer thickness of the doped HIL.

### 3.2 Injection Layer Doping and Device Conductivity

#### Current Voltage Characteristics

**FIGURE 4:**
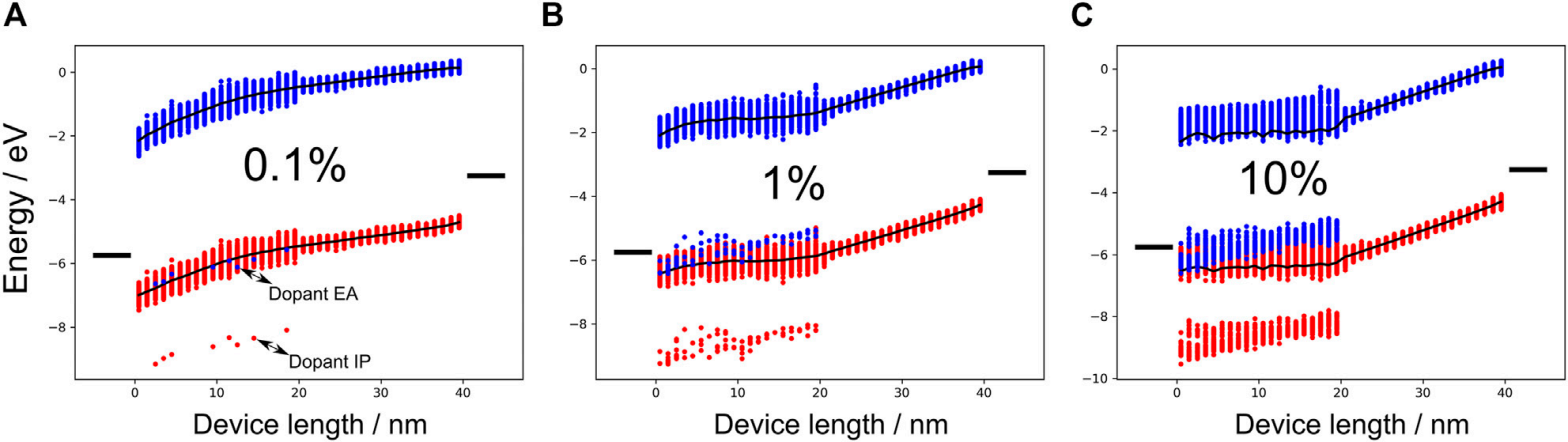
Energy diagrams of the test device for conductivity simulations. The black bars on the left of each panel represents the Fermi level of the electrodes. The red and blue dots illustrate the IP and EA, respectively. With a doping concentration of 0.1% **(A)**, the majority of red (blue) dots represent the host IPs (EAs). The dopant IPs and EAs are explicitly annotated. At such small doping concentrations, no Fermi level alignment is achieved. With a doping concentration of 1% **(B)**, the injection barrier is significantly smaller compared to the low doping case. Fermi level alignment is accomplished at a large doping concentration of 10% **(C)**.

**FIGURE 5:**
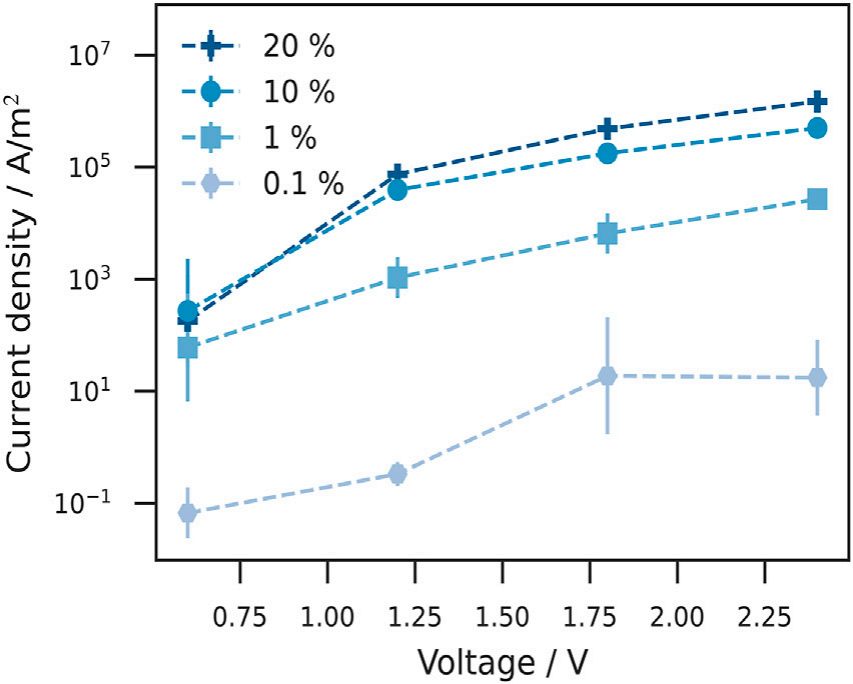
Current voltage characteristics (*J*–*V*-curve) at different doping concentrations. The current density is plotted against the applied voltage. At a low doping concentration 0.1% the current density is significantly smaller than for 1% doping concentration. Higher doping concentrations (10%) lead to an further increase of the current density. The difference between the 10 and 20% JV-curves is relatively small, suggesting saturation of the doping effect on the conductivity. The results here are in line with the energy diagrams in Figure 4.

In the second part of this work, we investigated how doped injection layers affect the transport properties in OLED devices. For this purpose we use a modified device with the hole blocking layer replaced by a hole transport layer (HTL) with the same energy levels as the host material in the doped HIL and a small energetic disorder of $\sigma_{\text{H}}^{\text{IP}}=$ 0.07  eV, a common value for good hole transport materials (Aydin and Yavuz, 2021). Figure 4 shows the energy diagram of this device with an applied electric field of 0.06 V nm^−1^ corresponding to an applied voltage of 2.4 V. With a doping concentration of 0.1% (Figure 4A), no Fermi level alignment is achieved which can be seen by the large injection barrier of the anode and doped HIL. As already shown in Figures 2, 3, larger doping concentrations (Figures 4B,C) allow the reduction of the injection barrier until Fermi level alignment is reached. Another doping induced effect is where in the device the applied voltage drops. Depending on the doping concentration, most of the applied voltage drops either in the HIL or HTL. If the doping concentration is high enough, no voltage drop occurs in the injection layer, since the field is compensated for by the newly acquired free charge carriers. As a consequence, the applied voltage must drop in the neighboring insulation layer. At a doping concentration of 0.1% (see Figure 4A), the energy cross section in the doped HIL has a strong slope, indicating a voltage drop here. This slope is strongly reduced at 1% doping concentration and the voltage drop in the HTL increases (see Figure 4B). At a doping concentration of 10% the energy levels in the doped HIL are flat and the entire voltage drops in the HTL (see Figure 4C). Mainly, we are interested in the current density–voltage characteristics (*J*–*V* curve) and their dependence on the doping concentration. The current density is computed by multiplying the average drift velocity of the charge carriers and dividing it through the device cross section. Figure 5 shows the *J*–*V* curve at different doping concentrations. The current density increases by several orders of magnitude from 0.1 to 1%. With doping concentrations of 1–10% the increase in current density is less strong. For 10–20%, there is only a minor increase which indicates a saturation of the doping induced impact on the current density. In the work of Murat Mesta et al. (2013), the doped injection layers were approximated by electrodes with the appropriate work functions. For large doping concentrations, we carried out simulations where the doped injection layer was replaced by an effective anode with a work function equal to the ionization potential of the host material. The results can be seen in the Supplementary Figure S1. Even at very large doping concentrations of 49%, replacing the doped injection layer with an effective anode leads to an overestimation of the current density by a factor of 2–5. Going towards doping concentrations more relevant in experiment, the effective anode leads to an overestimation of the current density by a factor of 20–100 and an increasing deviation in field-dependence.

#### Current Dependence of the Host IP at Different Doping Concentration

**FIGURE 6:**
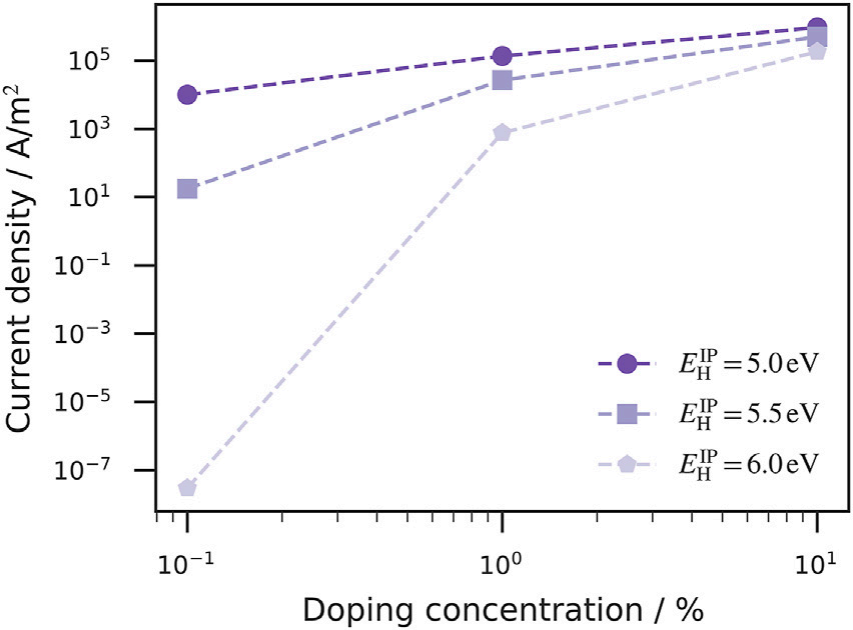
Current density as a function of the doping concentration and host ionization potentials (IP). The relation between the IP of the host materials and the anode workfunction determine the initial hole injection barrier. At small doping concentrations (0.1%) the current density for the host material with $E_{\text{H}}^{\text{IP}}=$ 6.0 eV is very small compared to the current densities of the devices with other host materials ( $E_{\text{H}}^{\text{IP}}=$ 5.0–5.5 eV). A doping concentration of 1% increases the current density of the device with $E_{\text{H}}^{\text{IP}}=$ 6.0 eV by nine orders of magnitude. The current enhancing effect is much weaker for the devices with lower initial injection barriers. The host materials IP plays a minor role for a large doping concentration (10%) due to achieved Fermi level alignment even for the $E_{\text{H}}^{\text{IP}}=$ 6.0 eV host material.

We have already discussed that Fermi level alignment can also be achieved for host materials that have a large IP when the doping concentration is sufficiently high (see Figure 2). The impact on device conductivity has an even greater importance for practical work, which is why the relationship between the current density, ionization potential of the host material and doping concentration was investigated here. For a given electrode workfunction, the host IP determines the initial hole injection barrier, before Fermi level alignment takes place due host/dopant ionization. In Figure 6, the current density is plotted against the doping concentration at different host IPs. At a low doping concentration (0.1%), the current density for host materials with smaller IP ( $E_{\text{H}}^{\text{IP}}=$ 5.0–5.5 eV) is eight to ten orders of magnitude larger than for the host with $E_{\text{H}}^{\text{IP}}=$ 6.0 eV. At a doping concentration of 1%, the enormous discrepancy between the current densities becomes much smaller. With a further increase of the doping concentration, the host IP no longer plays a role, since the initial injection barriers were almost completely eliminated for all three cases. It is particularly worth pointing out that for the host material with $E_{\text{H}}^{\text{IP}}=$ 6.0 eV (large initial injection barrier 1.5 eV), the current density could be increased by approximately 12 orders of magnitude by increasing the doping concentration from 0.1 to 10%. At these large doping concentrations, high current densities are achieved regardless of the host material used. The significant impact on the current density stems from two effects: 1) the increase of mobile charge carriers due to doping activation and 2) the resulting reduction of the hole injection barrier.

## 4 Conclusion

Doping of charge injection layers is an important step to control and improve overall OLED device performance. Computer-aided methods, such as the kMC model introduced here can accelerate the search for ideal host/dopant pairs and optimal device architecture. kMC methods struggle in modelling doped injection layers, because the computational effort scales badly with the number of carriers. Doped injection layers have a large number of carriers, leading to many fast processes due to the high conductivity in efficiently doped materials. For the simulation of realistic multi-layer OLEDs, most of the kMC steps are required for the doped injection layer, rendering explicit kMC simulations of full-stack devices numerically unfeasible. One way to overcome this problem is to treat doped injection layers as an effective electrode. However, this approximation is not suitable for accurate simulations of the current density, which we have shown in Supplementary Figure S1. Here we report on kMC simulations of the doped hole injection layer in a cubic, two-layer device that enable us to investigate the performance of the injection layer as a function of the doping concentration, the ionization potential of the host material, and the thickness of the layer.

One particular important aspect is the effect of doping the injection layer and the injection barrier on Fermi level alignment. Using the device shown in Figure 1 we could vary a wide range of critical parameters, such as host IP or dopant concentration to provide insights into the interplay of materials and layer configurations. In fabricated devices, the injection barrier is determined by the selection of the host material. Even with this parameter predetermined, this simulation can help optimize the doping concentration for Fermi level alignment and charge balance in the emissive layer.

Our results show that our kMC model is conceptually well suited to study the influence of doped injection layers on the device properties. We have shown in Figure 2 that by increasing the doping concentration, Fermi level alignment can be achieved even with large host IPs. In addition, we observed that a large layer thickness (with constant doping concentration) fosters Fermi level alignment. The effect of doping on current density is of particular practical interest. We could demonstrate that increasing the doping concentration can cause the current density to increase by many orders of magnitude (see Figure 5).

The simulated systems can be extended from cubic to realistic structures (Neumann et al., 2013), but we note that this approximation is less severe than one might think, because the off-diagonal disorder is captured by the distribution of hopping matrix elements. When applied to novel materials, accurate EAs and IPs can be obtained using ab-initio calculations (Armleder et al., 2021) and the Coulomb interaction of host/dopant pairs can be computed quantum-mechanically (Symalla et al., 2020a). In combination with an ab-initio parametrization, this work can help to accelerate computational screening for ideal host/dopant materials in doped injection layers and optimization of material composition and layer arrangements. In crystalline organic semiconductors the description of charge transport as sequential hopping processes may be invalidated by delocalization effects, which can occur especially at high doping concentrations or materials with high mobilities (Troisi, 2011; Oberhofer et al., 2017). Mixed quantum-classical methods (Wang et al., 2015; Stafström, 2010) might be used to take into account these effects.

In order to make kMC simulations of realistic many-layer OLEDs feasible, it is necessary to treat the doped injections layer in a special way. Treating the doped injection layer as an effective anode is thus not a general solution to the problem of kMC models when simulating entire OLED stacks, so more sophisticated effective models of the doped injection layer may solve the simulation time issue more precisely.

## Data Availability

The raw data supporting the conclusions of this article will be made available by the authors, without undue reservation.
