# Supplementary material for: Systematic kMC Study of Doped Hole Injection Layers in Organic Electronics
Source: Front Chem. 2022 Jan 18;9:809415. doi: 10.3389/fchem.2021.809415 (PMC8804170; doi:10.3389/fchem.2021.809415)
Supplement: Supplementary file 1 [file Table1.PDF]

Supplementary Material

1 SUPPLEMENTARY TABLES AND FIGURES

Table S1. Simulation parameters

Parameter	Value
Simulation box size L_y	$15\,\mathrm{nm}$
Simulation box size L_z	$15\,\mathrm{nm}$
Periodic boundary conditions $[x$ (transport axis)$, y, z]$	[No, Yes, Yes]
Dielectric constant in all organic layers ϵ_r	4.0
Insulator IP E_I^IP	$8.0\,\mathrm{eV}$
Insulator EA E_I^EA	$1.0\,\mathrm{eV}$
Electrode-Organic coupling for hole injection J_i^inj	$1.235 \times 10^{-22}\,\mathrm{eV}$
Constant factor in electronic coupling in Marcus expression j_0	$4.807 \times 10^{-22}\,\mathrm{eV}$
Coupling decay length a_0	$0.1\,\mathrm{nm}$
Temperature T	$300\,\mathrm{K}$
