# Supplementary figures and images for: Systematic kMC Study of Doped Hole Injection Layers in Organic Electronics
Source: Front Chem. 2022 Jan 18;9:809415. doi: 10.3389/fchem.2021.809415 (PMC8804170; doi:10.3389/fchem.2021.809415)

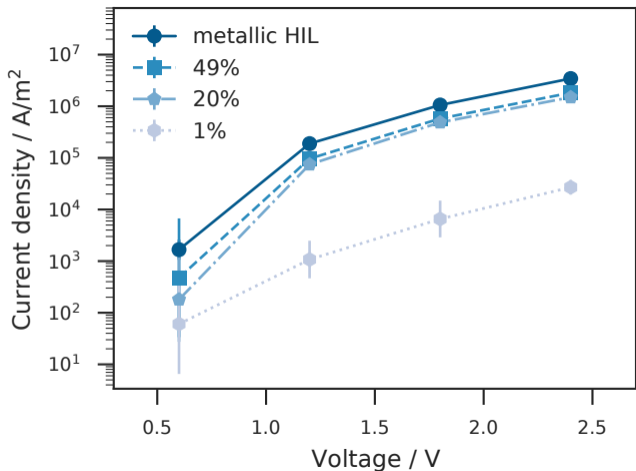

Supplement: Supplementary file 2 [file Image1.PDF]
